# Neonatal scrotochesis following artificial rupture of membrane in breech delivery

**DOI:** 10.1016/j.eucr.2025.103062

**Published:** 2025-05-05

**Authors:** Abdelrahman S. Elnour, Alaa Algurashe, Selma Karsani, Amal Alfatih, Moataz Idriss, Omer Ibrahim

**Affiliations:** a Specialist Pediatric Surgeon, Sennar Teaching Hospital Sudan, Sudan; b Pediatric Surgery Resident, Sudan Medical Specialization Board, Sudan; c Medical Officer, Sennar Teaching Hospital Sudan, Sudan

## Abstract

Birth trauma refers to structural or functional impairments in newborns caused by mechanical forces during labour and delivery, with breech presentation significantly increasing the risk of neonatal injuries. Scrotochesis, a disruption of the scrotal wall, is a rare complication associated with vaginal breech birth. We report a neonate who presented with scrotochesis following artificial rupture of membranes (ARM) during a term breech delivery. This case highlights the potential risks associated with breech deliveries and obstetric interventions, emphasizing the importance of careful monitoring and gentle manoeuvres to prevent perineal and scrotal trauma in neonates.

## 1 Introduction

Birth trauma refers to structural or functional impairments in newborns caused by mechanical forces during labour, delivery, or resuscitation, with outcomes ranging from minor superficial injuries to severe morbidity and mortality.^1^, ^2^, ^3^

The incidence of birth trauma varies globally, with higher rates reported in low- and middle-income countries (LMICs) compared to high-income countries. Key risk factors include operative vaginal delivery, fetal malpresentation, prolonged labour, macrosomia, nulliparity, inadequate antenatal care, and maternal comorbidities.^1^, ^2^, ^3^, ^4^, ^5^

Breech deliveries occur in 3–4 % of births and are associated with a significantly higher risk of birth trauma, especially during Vaginal Breech Birth (VBB), where birth injuries are twice as common. VBB poses increased risks of maternal morbidity, neonatal mortality, birth asphyxia, and birth injuries compared to cesarean delivery.^5^, ^6^, ^7^

Neonatal perineal and scrotal injuries are extremely rare with limited case reports documenting such occurrences.^6^, ^7^, ^8^, ^9^, ^10^, ^11^, ^12^ Scrotochesis is a rare congenital or traumatic condition characterized by a defect or disruption of the scrotal wall, leading to exposure of the underlying structures, such as the tunica vaginalis and testis.^11^, ^12^, ^13^

We report a neonate who presented with scrotochesis following artificial rupture of membranes (ARM) during a term breech delivery. This case highlights the potential risks associated with breech deliveries and obstetric interventions, emphasizing the importance of careful monitoring and gentle manoeuvres to prevent perineal and scrotal trauma in neonates. This manuscript was prepared following the CARE guidelines (https://www.care-statement.org).

## 2 Case presentation

A term male neonate weighing 3.5 kg was admitted 3 h post-delivery following a home vaginal breech birth. The mother, a 25-year-old para 2, had limited antenatal care, attending only one visit where an ultrasound confirmed breech presentation. The delivery was conducted by a trained midwife, who performed (ARM) to expedite prolonged labour. During the procedure, direct trauma to the scrotum occurred, resulting in a left hemiscrotal injury.

**Fig. 1:**
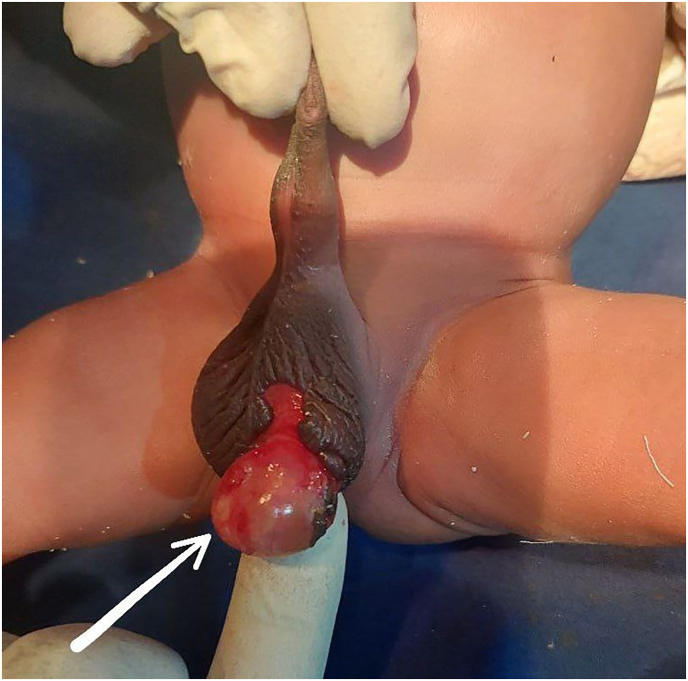
Left hemiscrotum degloving injury with exposed tunica vaginalis.

**Fig. 2:**
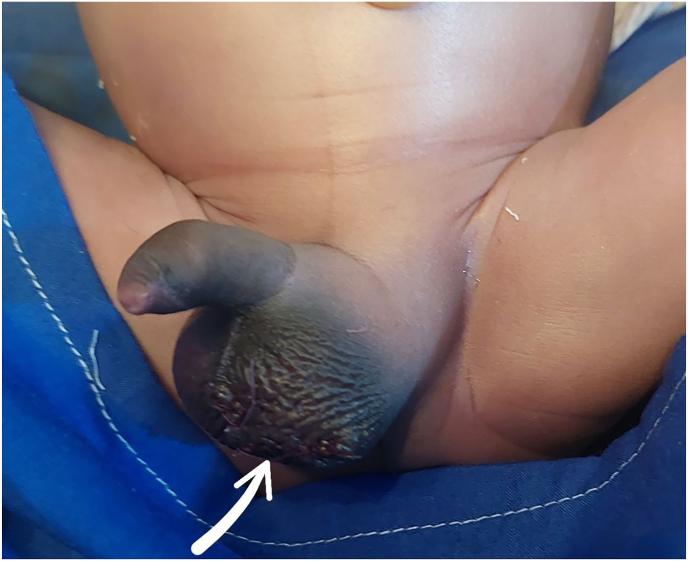
Immediate postoperative image following left hemiscrotum exploration and reconstruction.

On admission, the newborn was pink, active, and without dysmorphic features or signs of distress. Cardiovascular, respiratory, abdominal, and inguinal examinations were unremarkable. The right hemiscrotum, penis, and anus were intact. However, the left hemiscrotum had a degloving injury with exposed tunica vaginalis, though there was no associated bruising, swelling, or skin loss (Fig. 1). Emergency surgical exploration under general anesthesia confirmed a left hemiscrotal degloving injury caused by the ARM, with an intact tunica vaginalis and a viable testis. Primary wound closure was performed (Fig. 2). The postoperative course was uneventful, and the neonate was discharged in good condition.

## 3 Discussion

Birth injuries include a spectrum of injuries ranging from minor to major injuries resulting from mechanical forces during labour and delivery. Unlike birth defects or malformations, birth injuries can be easily distinguished through focused clinical assessment. Although birth trauma rates have decreased due to improved obstetrical techniques and increased cesarean deliveries, it remains a significant public health issue in (LMICs), with high incidence and associated mortality and morbidity.^2^^,^^5^^,^^10^

Breech presentation is a high-risk situation that requires hospital management, as home deliveries significantly increase maternal and neonatal risks. In developing countries, poor antenatal care, lack of awareness, a shortage of skilled birth attendants, and delayed referrals further exacerbate these challenges, leading to a higher risk of complications for both the mother and baby.^1^, ^2^, ^3^, ^4^, ^5^

Obstetric manoeuvres and interventions, including (ARM), should be performed with caution and by a well-trained medical professional to decrease the risk of injuries minimizing potential complications during delivery as observed in our patient.^2^^,^^3^^,^^10^

Genital injuries are more common in vaginal breech deliveries and can involve the scrotum, testicles, penis, buttocks, anus, labia, and perineal body. These injuries are more frequently observed in singleton infants born after 40 weeks of gestation and with birth weights exceeding 2.5 kg. Factors such as repeated vaginal examinations, instrumentation, or forceful extraction of the breech may contribute to scrotal injuries.^2^^,^^5^^,^^8^

Careful neonatal assessment and timely emergency exploration lead to excellent outcomes, especially in cases without skin loss or testicular injury.^11^, ^12^, ^13^

## 4 Conclusion

Neonatal scrotochesis is an exceptionally rare complication of vaginal breech delivery. This case underscores the risks associated with home deliveries, particularly in the absence of adequate antenatal care and skilled obstetric management. It highlights the critical need for improved antenatal screening for malpresentation and timely obstetric interventions to minimize birth trauma and enhance neonatal outcomes, especially in resource-limited settings.

## CRediT authorship contribution statement

**Abdelrahman S. Elnour:** Writing – review & editing, Writing – original draft, Supervision, Conceptualization. **Alaa Algurashe:** Writing – original draft. **Selma Karsani:** Resources, Data curation. **Amal Alfatih:** Resources, Data curation. **Moataz Idriss:** Investigation, Data curation. **Omer Ibrahim:** Software, Data curation.

## 5 Statements

1) Informed consent was obtained from the patient's guardian.
2) All authors attest that they meet the current ICMJE criteria for Authorship.

## Funding

None.

## Conflicts of interests

There are no conflicts of interest to declare.
